# Measuring neuropsychiatric symptoms in patients with early cognitive decline using speech analysis

**DOI:** 10.1192/j.eurpsy.2021.2236

**Published:** 2021-10-13

**Authors:** Alexandra König, Elisa Mallick, Johannes Tröger, Nicklas Linz, Radia Zeghari, Valeria Manera, Philippe Robert

**Affiliations:** 1 Stars Team, Sophia Antipolis, Institut National de Recherche en Informatique et en Automatique (INRIA), Valbonne, France; 2 Clinical Research, ki:elements, Saarbrücken, Germany; 3 CoBTeK (Cognition-Behaviour-Technology) Lab, FRIS-University Côte d’Azur, Nice, France

**Keywords:** apathy, depression, mild neurocognitive disorders, neuropsychiatric symptoms, speech analysis, vocal parameters

## Abstract

**Background:**

Certain neuropsychiatric symptoms (NPS), namely apathy, depression, and anxiety demonstrated great value in predicting dementia progression, representing eventually an opportunity window for timely diagnosis and treatment. However, sensitive and objective markers of these symptoms are still missing. Therefore, the present study aims to investigate the association between automatically extracted speech features and NPS in patients with mild neurocognitive disorders.

**Methods:**

Speech of 141 patients aged 65 or older with neurocognitive disorder was recorded while performing two short narrative speech tasks. NPS were assessed by the neuropsychiatric inventory. Paralinguistic markers relating to prosodic, formant, source, and temporal qualities of speech were automatically extracted, correlated with NPS. Machine learning experiments were carried out to validate the diagnostic power of extracted markers.

**Results:**

Different speech variables are associated with specific NPS; apathy correlates with temporal aspects, and anxiety with voice quality—and this was mostly consistent between male and female after correction for cognitive impairment. Machine learning regressors are able to extract information from speech features and perform above baseline in predicting anxiety, apathy, and depression scores.

**Conclusions:**

Different NPS seem to be characterized by distinct speech features, which are easily extractable automatically from short vocal tasks. These findings support the use of speech analysis for detecting subtypes of NPS in patients with cognitive impairment. This could have great implications for the design of future clinical trials as this cost-effective method could allow more continuous and even remote monitoring of symptoms.

## Introduction

The amount of patients with cognitive decline and thus, the risk to develop dementia continues to rise as the population ages [1]. Neuropsychiatric symptoms (NPS) can be defined as behavioral noncognitive disturbances such as depression or agitation and are extremely common in dementia appearing in up to 98% of patients at one point during the disease [2]. It has been argued that NPS can appear relatively early even prior to dementia diagnosis, representing eventually an opportunity window for timely detection and treatment [3]. Since pathological changes seem to occur in the brain often before the onset of clinical dementia, the damages due to neurodegeneration may alter in a subtle manner emotion regulation and behavior as cognition starts to decline [4]. This suggests that underlying neurobiological mechanisms might underpin the appearance of these symptoms [5] contributing to an increased vulnerability and risk for conversion [6].

NPS can cause a great burden for patients and caregivers affecting severely disease course and quality of life [7]. Interestingly, even in cognitively normal subjects it has been reported that certain types of NPS may represent promising prognostic factors for predicting cognitive deterioration, especially the affective ones such as depression, apathy, and anxiety [8–10]. Anxiety seems to be an important predictor of amyloid pathology. The presence of apathy may increase the risk of conversion from mild cognitive impairment (MCI) to dementia [11] and a similar association is found with depression [12]. Differential utilities of NPS for even predicting dementia subtypes are currently intensively investigated [6] since it could lead to earlier tailored treatment.

Despite the immense clinical impact of NPS, these clinical dimensions remain insufficiently identified and therefore untreated. Evaluation and management of NPS remains challenging since its detection is not made systematically in current clinical practice in patients with cognitive decline [9], and these are often assessed only when family members bring their observation to the attention of health care professionals. However, there is evidence that if early treatment is provided, namely in the form of nonpharmacological intervention, it can be effective to reduce the severity of NPS [7], and even to slow down cognitive decline [11].

Current assessment methods rely mostly on clinical scales such as the neuropsychiatric inventory (NPI) [13], which in turn depends heavily on the informants’ objective reporting ability [14]. The clinician rating may be influenced as well by caregivers’ personal experiences, and by limited access to reliable information. This is why clinicians and researchers are looking for ways to complement classical assessment scales with more objective indexes, which are able to detect subtle variations in the presentation of NPS as well as its different subtypes [15, 16].

Mobile technologies may be a promising solution for detecting and monitoring objectively fine-grained changes in behavior and thus, the appearance of NPS in a continuous manner [17]. The use of digital biomarkers which are measures of behavioral data collected by means of digital devices are increasingly studied in patients with cognitive decline as a potential alternative assessment approach [18]. One promising avenue lies in recent advances in computational linguistics and language processing that have led to the use of automatic speech analysis in the assessment of various clinical manifestations [19, 20] making it potentially useful for assessing different NPS.

Until now, it has been clearly demonstrated that changes in speech and language patterns can be indicative for cognitive decline due to neuropathological processes. Different affective states including depression, anxiety, and apathy alter as well mechanisms involved in speech production, namely variations in muscle tension and tonality which can affect prosody and the quality of speech. Reduced or increased muscle tension will influence vocal tract dynamics as well as articulation behavior [21] which may be today with recent advances in speech analysis technologies be easily detectable.

In depression, which is characterized by persistent sadness and anhedonia, accompanied by disturbed sleep/appetite, fatigue, and even suicidal ideation, it is notable by ear that patients show a reduced speech rate and prosody spectrum and sound rather monotonous which could serve as markers, if objective measurements can quantify these observations [22]. Until now, several groups investigated the use of automatic analysis of speech as an additional assessment tool with an extensive review published by Cummins et al. [21] outlining the interest of using speech as a key objective marker for disease progression.

Prosodic, articulatory, and acoustic features of speech seem affected by depression severity and thus can easily be identified and used for continuously monitoring patients. With a considerable overlap of symptoms between depression and apathy, namely the lack of interest and goal-oriented behavior, we anticipate similar results when applying speech technology methods to apathy with a slightly different pattern in regards to emotionally triggered speech.

In anxiety, which is characterized by excessive fear or worrying, many studies found a significant increase in mean fundamental frequency (*f*0) [23]. Jitter and shimmer were also significantly higher in anxious patients [24, 25]. In a previous study, we showed that certain speech characteristics extracted over the phone correlated with stress levels in both genders; mainly, spectral (i.e., formant) features, such as the mel-frequency cepstral coefficient (MFCC), and prosodic characteristics, such as the fundamental frequency, appeared to be sensitive to stress [26].

In apathy, which is characterized by lack of motivation, decreased initiative, and emotional indifference, we demonstrated that certain paralinguistic features, namely temporal aspects of speech as well as prosodic characteristics correlate significantly with levels of symptom severity [27].

Hence, given the increasingly important role of NPS in early dementia diagnosis [28], it seems worthwhile investigating the additional value of this method further for differential diagnosis of these most frequently found NPI symptoms. Therefore, the purpose of this study is to determine whether automatic speech analysis and the automatic extraction of speech features can be useful for the early detection of specific NPS—namely depression, apathy, and anxiety—in patients with mild neurocognitive disorders.

## Materials and Methods

### Participants

All participants were recruited through the Memory Clinic located at the Institut Claude Pompidou in the Nice University Hospital. A total of 141 patients aged 65 or older with mild neurocognitive disorder according to the Diagnostic Statistical Manual 5 (DSM-5) [29] were included in this study. For this, the presence of cognitive decline in memory and/or executive function with or without interference with performance in activity of daily living was required based on previously performed evaluations. Patients coming to the Memory clinic underwent a clinical assessment including, among others, the Mini-Mental State Examination (MMSE) [30] and the NPI interview [13].

### Study procedure

Speech features vary naturally between males and females. These differences have been leveraged in gender classification through speech analysis based on pitch and formant frequencies [31], Harmonic to Noise ratio [32], and linear predictive components and MFCC [33]. Previous work found differences in speech depending on gender in the effects of apathy [34], as well as depression and the effectiveness of classifiers for its detection [35]. This is why this study considers males and females separately resulting in two experimental groups consisting of 92 females and 49 males.

Participants were all native speakers of French and excluded if they had any major auditory or language problems, history of head trauma, loss of consciousness, psychotic or aberrant motor behavior, or history of drug abuse. Written informed consent was obtained from all subjects prior to the experiments. The study was approved by the Nice Ethics Committee (ELEMENT ID RCB 2017-A01896-45, MoTap ID RCB 2017-A01366–47) and was conducted according to the Declaration of Helsinki.

### Speech task (positive and negative story)

Free and natural speech tasks are capable of eliciting emotional reactions (or a lack thereof) by asking to describe events that triggered recent affective arousal. Affective arousal is expected to impact speech in NPS since simple vocal exercises or reading tasks do not allow to capture the acoustic effects of alterations in affective states [21]. Using emotional induced free speech tasks allows for a greater range of emotional effects such as describing events that have aroused significant emotions [36]. Therefore, the participants were asked to: (a) talk about a positive event in their life and (b) to talk about a negative event in their life. Instructions for the speech tasks (“Can you tell me in one minute about a positive/negative event in your life?”) were prerecorded by one of the psychologists and played from a tablet computer ensuring standardized instruction over both experiments. The answers were recorded with the tablet’s internal microphone.

### Processing of speech data

Audio features were extracted directly and automatically from the speech signal. This form of speech analysis does not consider the semantic content of what a participant said, thus increasing the applicability of results in a clinical scenario, as no prior processing, such as transcription of what has been said, is required. For each speech task (positive and negative story), features were extracted separately from different main areas: *temporal* features including measures of speech proportion (e.g., length of pauses and length of speaking segments), the connectivity of speech segments and general speaking rate; prosodic, relating to long-time variations in perceived stress and rhythm in speech. *Prosodic* features also measure alterations in personal speaking style (e.g., perceived pitch and intonation of speech); *formant* features represent the dominant components of the speech spectrum and carry information about the acoustic resonance of the vocal tract and its use. These markers are often indicative of problems with articulatory coordination in speech motor control disorders (ref Sapir); *source* features relate to the source of voice production, the airflow through the glottal speech production system. *Spectral* features characterize the speech spectrum; the frequency distribution of the speech signal at a specific time instance information in some high dimensional representation. These features operationalize irregularities in vocal fold movement (e.g., measures of voice quality). An overview and explanation of the extracted speech features can be found in Table 1.

**Table 1.** Overview and explanation of extracted speech features.

Speech features	Explanation	Category
Speech ratio	Prosodic feature for verbal fluency from speech to nonspeech proportion in the audio	Temporal
Speech interval	Speech segments uninterrupted by pauses between syllables to measure speech production efficiency	Temporal
Total phonation time	Total time duration of all words across all sentences	Temporal
Number of pauses	Total number of pauses longer than a time threshold between the syllables	Temporal
Harmonics to noise ratio	Ratio between periodic and nonperiodic components of speech which reflects voice quality	Source
Sound to noise ratio	Ratio between the power of speech signal and the power of background noise that reflects voice quality	Source
Jitter	Jitter is a measure of random perturbation in signal periodicity more representative when examining long vowels. Jitter calculation is based on relative jitter	Source
Espinola zero crossing metric	Measure for the rate at which speech signal crosses the zero reference and its deviation from the reference	Source
Average amplitude change	Variation of the signal amplitude over time	Source
Amplitude mean absolute value	Variation of the signal amplitude over time without change direction	Source
Amplitude third moment	Skewness of the signal amplitude over time	Source
Amplitude fourth moment	Kurtosis of the signal amplitude over time	Source
Max amplitude	Measure for the maximum disturbance of the air caused by the speech signal	Source
Mean power	Mean power transmitted by the signal	Source
Total power	Total power transmitted by the signal	Source
*F*_0_ mean	Mean value of the fundamental frequency in voiced parts of the audio. Fundamental frequency or pitch quantifies speech signal’s periodic components for speech production assessment	Prosodic
*F*_0_ standard deviation	Standard deviation of fundamental frequency in voiced parts of the audio	Prosodic
Average MFCC	Decomposition of MFCC into a range of spectrum coefficients 1 through 20 MFC represents the short-term power spectrum of a sound	Spectral
Deltas	First derivative of the average MFCC values that presents the change in the power spectrum	Spectral
Delta deltas	First derivative of the average MFCC values that presents the rate of change in the power spectrum	Spectral
Peak frequency	Frequency of the maximum power value in signal frequency spectrum	Spectral
Power spectrum ratio	Power of the most powerful frequency relative to all other frequencies	Spectral

Abbreviation: MFCC, mel-frequency cepstral coefficient.

### Statistical analysis

Statistical analysis was run using R software (software version 3.4.02). QQ-plots as well as Shapiro–Wilk tests indicated a violation of the normal distribution for the depression scores. Therefore, we used Spearman rank correlations to test whether speech as well as transcript data was correlated with NPI scores (only for the items apathy, depression, and anxiety). We applied the Benjamini–Hochberg procedure for each of the feature categories when correcting for multiple comparisons. Three variable groups were excluded from this analysis. MFCCs, Delta, and Delta Delta values were not considered in group comparisons, as they do not have directly human understandable explanations and would therefore provide little insight. These variables were included in the machine learning experiments, as explainability is not required.

To predict NPI scores, we train different regression models and evaluate their performance using MAE. Regression models are trained including support vector regression (SVR) and Lasso (Linear Regression with L1 regularization). Implementations were provided by the scikit-learn python framework. Features were normalized by subtraction of their mean and division through their standard deviation. Because of the small data set size a separate validation/test set could not be used. Instead, Leave-One-Out-Cross-Validation was employed. This is a method where *N* different models are evaluated: train a model on *N* − 1 observations and test it on one observation. The model is evaluated for every held-out observation. The final result is then calculated by taking the mean of all the individual evaluations.

## Results

**Figure 1.**
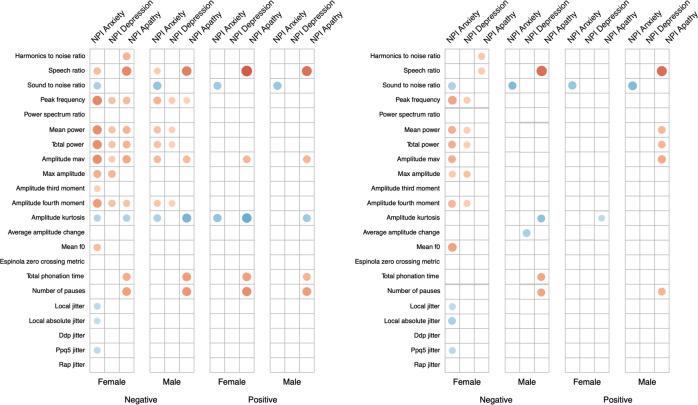
Plot between (left) Spearman rank correlations and (right) spearman rank partial correlations corrected for Mini-Mental State Examination (MMSE), between audio features and neuropsychiatric inventory (NPI) subscales, separated by gender and voice task. Only significant correlations are reported. Absolute value of correlation is reflected in the size and color (positive correlations in blue; negative correlations in red) of the dot.

A total of 141 participants, of which 92 were female and 49 were male, have been included in the analysis. Demographic and clinical information split by gender is available in Table 2. Both groups had an average age around 75 and showed on average some signs of MCI, with a mean MMSE of around 24. The male group scored significantly higher on the apathy domain of the NPI. Correlations between the NPI subscales and the MMSE showed significant correlations for both groups (see Table 3). MMSE was significantly correlated with the NPI apathy subscale for both groups, while only females also showed significant correlations between the anxiety subscale and the MMSE. Both normal and partial correlations, corrected for MMSE, of speech variables and NPI subscales by gender group and speech task are displayed in Figure 1 (a detailed listing of correlations is provided in Supplementary Tables S1 and S2).

**Table 2.** Demographic data for included participants, split by gender.

	Females	Males
*N*	92	49
Age	75.01 (7.88)	76.08 (6.75)
NPI anxiety	1.84 (2.64)	1.86 (2.67)
NPI depression	0.85 (1.90)	0.84 (1.69)
NPI apathy	1.64 (2.74)	3.37 (3.81)
NPI total	7.10 (9.23)	9.82 (8.96)
MMSE	24.01 (4.95)	23.69 (4.34)

*Note:* Mean and standard deviation are reported.

Abbreviations: MMSE, Mini-Mental State Examination; NPI, neuropsychiatric inventory.

**Table 3.** Spearman rank correlations between MMSE and NPI subscales for females and males.

NPI score	Females		Males	
	Correlation	*p*-value	Correlation	*p*-value
Anxiety	−0.335	<0.01	−0.268	>0.05
Depression	−0.148	>0.05	−0.163	>0.05
Apathy	−0.584	<0.001	−0.486	<0.001

Abbreviations: MMSE, Mini-Mental State Examination; NPI, neuropsychiatric inventory.

The *Sound to noise ratio* shows significant correlations with the NPI anxiety subscale for both genders and tasks, suggesting that the higher the sound to noise ratio, the stronger symptoms of anxiety are present. This effect is stable after correcting for the influence of MMSE. Before and after correcting for MMSE, the *Speech Ratio* shows significant correlations with the NPI apathy subscale for both genders and across both tasks, indicating that less speaking during the recording is associated with higher levels of apathy. The same is visible for the *Total Phonation Time* and *Number of Pauses*, but effects are not sustained after correcting for MMSE using partial correlations. The *Amplitude Kurtosis* shows significant correlations with the anxiety and apathy NPI subscales, which are not retained in the partial correlation.

Correlations between all NPI scales and measures of power (*Mean power* and *Total power*) are visible for females in the negative story. When correcting for the influence of MMSE, only weaker correlations with the anxiety and depression subscales remain. Performance of trained machine learning models is listed in Table 4 as the MAE of predictions. The baseline for each subscale and gender is reported. For each subscale and gender, a model could be trained that outperforms this baseline by at least 0.3 points on the NPI scale. Features selected by the machine learning models are available in Supplementary Table S3.

**Table 4.** Mean absolute error of regression methods (linear regression L1 penalization and SVM) and of the baseline, for males and females separately.

	Male			Female		
	Baseline	Linear regression L1 penalization	SVM	Baseline	Linear regression L1 penalization	SVM
NPI anxiety	2.05232	2.05232	**1.68202**	2.17857	2.22331	**1.73591**
NPI depression	1.26708	1.26708	**0.89701**	1.08078	1.08078	**0.86314**
NPI apathy	2.13903	**1.84431**	**1.66386**	3.22024	**2.84111**	3.37315

*Note:* Results better than baseline are marked in bold.

Abbreviation: NPI, neuropsychiatric inventory.

## Discussion

Identifying early signs of different NPS by the means of objective measurement tools could ultimately lead to better management and treatment. This is particularly important in patients with mild neurocognitive disorders. Indeed, the assessment of NPS is not systematically performed in clinical practice, despite the fact that they represent risk factors for dementia conversion, and thus are increasingly becoming targets of clinical trials [37, 38]. As in the early stages of neurocognitive disorders NPS do not interfere substantially with activities of daily living, patients, and caregivers may not report them unless prompted, which suggests the need for noninvasive, objective, and reliable measures that can help detect subtle changes in NPS.

The results of the present study show that certain changes in spontaneous speech characteristics seem associated with specific NPS. Namely, apathy correlates with temporal aspects of speech such as *speech ratio*, which means that patients with more severe apathy, speak slower and less. This is consistent with the diagnostic criteria for apathy [39], which identified reduction of self-initiated verbal production as one of the core apathy dimensions. Similarly, we found previously strong correlations between this type of features (sound duration, syllable count, etc.) and subdomains of the apathy inventory [27].

In turn, anxiety correlates with voice quality features such as *Sound to noise ratio.* These results are even mostly consistent between male and females. Importantly, these correlations between apathy and anxiety and the distinct speech variables were still significant after correcting for cognitive impairment, meaning that these specific speech features may be relevant for assessing NPS in patients with different degrees of cognitive deterioration, and do not capture simply cognitive decline. As the presence of NPS increases with cognitive decline progression [28], it is important to control for the degree of cognitive decline to verify if speech features are relevant specifically to detect NPS. The fact that we did not find any strong correlation between speech features and depression might be due to the small variance on the depression NPI subscale in our sample.

Our findings are consistent with other research in which a significant increase in mean *f*0 (prosodic related feature) was detected in anxiety disorders [40–42]; however, we found this effect only in females during the negative storytelling. In another study, in which emotional responses were induced by watching videos, females showed higher emotional expressivity for negative stimuli, whereas males showed overall more intense emotional experiences, meaning that the differences in gender seem to depend on the specific emotion type [43].

Moreover, greater *F*0 has been reported mostly in males with social anxiety disorder compared to controls and only in females during in vivo social exposure [42]. This could explain why we found a difference only in the negative task since it may elicit a more emotionally loaded reaction. Jitter and shimmer were also significantly higher in patients with an elevated score on the NPI anxiety subdomain, which is in line with previous studies [24, 25].

Machine learning regressors were able to extract information from speech features and perform above baseline in predicting anxiety, apathy, and depression scores, despite NPS were globally of quite low intensity in our sample. When adding MMSE as a feature, only the model predicting apathy heavily relied on it, which may be partially due to the fact that apathy becomes more common with increased cognitive decline [44]. These results may have important clinical implications since for each subscale and gender, a model could be trained that outperforms this baseline by at least 0.3 points on the NPI scale.

The positive and negative storytelling were chosen as semistandardized free speech tasks, that still allow for production of natural speech, while limiting the scope/time and induce an emotional reaction in participants. This in contrast to related work measuring cognitive function from similar populations, that relied on more standardized tasks, such as describing an image or completing a verbal cognitive task.

Similar studies have shown as well that computed acoustic features can eventually be used to track changes in mental health states [45]. Indeed, speech analytics have been tested successfully in psychiatry for its use to predict treatment response in depressed patients [46] or changes in mood states in bipolar disorder [47]. As certain feature types seem to be associated with NPS, the outcome of this study may encourage a potential broader use of automatic speech analysis in other neurocognitive disorders for NPS detection. This could particularly improve the objectivity of first front-line assessment procedures and help overcome barriers that prevent timely access diagnosis and treatment.

In parallel to classical methods, such technologies can be used to establish objective, personalized baseline reference standards to design innovative clinical trials that assess the effectiveness of new treatments [48]. For instance, the use of wearable devices for additional measurements such as heart rate variability or locomotor activity are increasingly investigated for detecting behavioral abnormalities [49]. Combined with speech all these data could help reduce evaluation biases and provide richer understanding of variations in NPS on a day-to-day basis even before severity reaches a level of requiring intervention [50, 51]. In regards to the new rise of decentralized clinical trials, digital technologies will play a major role to complement traditional data acquisition enabling the redesign and adaptation of trials while still ongoing [52].

One limitation of our study is that patients in the sample showed a lot of variability in the MMSE score even if they all met the diagnosis criteria for mild cognitive disorder, which could have affected the results. In addition, patients had no severe NPS—rarely they scored above four, which is the original cut-off. However, taking into account the predictive value of behavioral symptoms [53], it is therefore important to investigate if the technology is able to detect subtle signs of symptoms since this may be even more of interest for early prevention strategies.

Another limitation is that we employed only the NPI scale for the assessment of NPS. Despite the fact that it is widely used and considered as a gold standard for neuropsychiatric assessment, the NPI is probably not the ideal scale for early screening, and to quantify fine changes. Recently, the Mild behavioral Inventory[54] has been proposed, that could better capture early signs of NPS, and thus may show stronger correlations with speech features in patients with mild NPS symptoms, as it is often the case for people with mild neurocognitive disorders. In addition, a larger study including more patients and more severe NPS symptoms should be replicated in order to validate the found speech markers.

## Data Availability

The data that support the findings of this study are available from the corresponding author upon reasonable request.

## Supplementary Materials

To view supplementary material for this article, please visit http://dx.doi.org/10.1192/j.eurpsy.2021.2236. click here to view supplementary material
