# Supplementary material for: Measuring neuropsychiatric symptoms in patients with early cognitive decline using speech analysis
Source: Eur Psychiatry. 2021 Oct 13;64(1):e64. doi: 10.1192/j.eurpsy.2021.2236 (PMC8581700; doi:10.1192/j.eurpsy.2021.2236)
Supplement: Supplementary file 1 [file S0924933821022367sup001.docx]

**Supplementary Material**

**Tables**

**TABLE S1.** Top 10 highest spearman rank correlations between audio features calculated per speech task and NPI subscales. Results are split up by gender.

(a) (Female; Anxiety)

| **Positive story** |  |  | **Negative story** |  |  |
| --- | --- | --- | --- | --- | --- |
| **Feature** | **Correlation** | **p value** | **Feature** | **Correlation** | **p value** |
| sound_to_noise_ratio | 0.3469641 | 0.0007031 | peak_frequency | -0.4433038 | 9.622e-06 |
| peak_frequency | -0.3012164 | 0.003526 | amplitude_mav | -0.4230408 | 2.655e-05 |
| amplitude_kurtosis | 0.2851256 | 0.005872 | total_power | -0.4223768 | 2.742e-05 |
| amplitude_mav | -0.2809288 | 0.006676 | mean_power | -0.4223768 | 2.742e-05 |
| total_power | -0.2690185 | 0.009513 | amplitude_fourth_moment | -0.3832605 | 0.0001627 |
| mean_power | -0.2690185 | 0.009513 | max_amplitude | -0.3171703 | 0.002066 |
| amplitude_fourth_moment | -0.2468373 | 0.01769 | sound_to_noise_ratio | 0.2830272 | 0.006262 |
| speech_ratio | -0.2232307 | 0.03244 | mean_f0 | -0.2781462 | 0.007262 |
| max_amplitude | -0.1895974 | 0.07027 | speech_ratio | -0.273535 | 0.008332 |
| total_phonation_time | -0.1268491 | 0.2282 | amplitude_kurtosis | 0.258846 | 0.01272 |

(b) (Male; Anxiety)

| **Positive story** |  |  | **Negative story** |  |  |
| --- | --- | --- | --- | --- | --- |
| **Feature** | **Correlation** | **p value** | **Feature** | **Correlation** | **p value** |
| sound_to_noise_ratio | 0.3396247 | 0.01697 | amplitude_kurtosis | 0.3433922 | 0.01571 |
| espinola_zero_crossing_metric | -0.1831235 | 0.2079 | sound_to_noise_ratio | 0.3172379 | 0.02635 |
| total_phonation_time | -0.1097501 | 0.4528 | amplitude_mav | -0.2490399 | 0.08442 |
| power_spectrum_ratio | 0.1057096 | 0.4697 | speech_ratio | -0.2163333 | 0.1354 |
| total_power | -0.09555357 | 0.5137 | number_of_pauses | -0.2162034 | 0.1357 |
| mean_power | -0.09555357 | 0.5137 | mean_f0 | -0.1889312 | 0.1936 |
| speech_ratio | 0.09511676 | 0.5156 | peak_frequency | -0.1708498 | 0.2405 |
| mean_f0 | -0.07978293 | 0.5858 | total_power | -0.164516 | 0.2586 |
| local_absolute_jitter | 0.06655989 | 0.6495 | mean_power | -0.164516 | 0.2586 |
| average_amplitude_change | -0.06585006 | 0.653 | total_phonation_time | -0.159711 | 0.273 |

(c) (Female; Depression)

| **Positive story** |  |  | **Negative story** |  |  |
| --- | --- | --- | --- | --- | --- |
| **Feature** | **Correlation** | **p value** | **Feature** | **Correlation** | **p value** |
| peak_frequency | -0.2291068 | 0.02804 | max_amplitude | -0.292862 | 0.004611 |
| amplitude_fourth_moment | -0.2163193 | 0.03835 | peak_frequency | -0.2623844 | 0.01151 |
| total_power | -0.2116438 | 0.04284 | amplitude_fourth_moment | -0.260786 | 0.01205 |
| mean_power | -0.2116438 | 0.04284 | total_power | -0.2544122 | 0.01439 |
| amplitude_mav | -0.1882766 | 0.07229 | mean_power | -0.2544122 | 0.01439 |
| max_amplitude | -0.1748197 | 0.09556 | amplitude_mav | -0.2281278 | 0.02873 |
| amplitude_third_moment | -0.1557183 | 0.1383 | local_jitter | 0.1580161 | 0.1325 |
| sound_to_noise_ratio | 0.1425711 | 0.1752 | ppq5_jitter | 0.1381954 | 0.1889 |
| mean_f0 | -0.1225804 | 0.2444 | rap_jitter | 0.1316518 | 0.211 |
| espinola_zero_crossing_metric | -0.0778772 | 0.4606 | ddp_jitter | 0.1316518 | 0.211 |

(d) (Male; Depression)

| **Positive story** |  |  | **Negative story** |  |  |
| --- | --- | --- | --- | --- | --- |
| **Feature** | **Correlation** | **p value** | **Feature** | **Correlation** | **p value** |
| average_amplitude_change | 0.2030018 | 0.1618 | average_amplitude_change | 0.2699959 | 0.06063 |
| harmonics_to_noise_ratio | 0.1820364 | 0.2106 | amplitude_fourth_moment | 0.1450946 | 0.3199 |
| local_jitter | -0.1503211 | 0.3026 | peak_frequency | 0.1181306 | 0.4189 |
| ppq5_jitter | -0.1214566 | 0.4058 | local_absolute_jitter | -0.1035796 | 0.4788 |
| local_absolute_jitter | -0.1152798 | 0.4303 | mean_f0 | 0.09952806 | 0.4962 |
| rap_jitter | -0.1086873 | 0.4573 | power_spectrum_ratio | 0.0969277 | 0.5076 |
| ddp_jitter | -0.1086873 | 0.4573 | local_jitter | 0.08112944 | 0.5795 |
| amplitude_fourth_moment | 0.1041735 | 0.4763 | ppq5_jitter | 0.08053552 | 0.5823 |
| total_phonation_time | -0.09591804 | 0.5121 | total_power | 0.07394301 | 0.6136 |
| max_amplitude | 0.09455202 | 0.5181 | mean_power | 0.07394301 | 0.6136 |

(e) (Female; Apathy)

| **Positive story** |  |  | **Negative story** |  |  |
| --- | --- | --- | --- | --- | --- |
| **Feature** | **Correlation** | **p value** | **Feature** | **Correlation** | **p value** |
| speech_ratio | -0.4649382 | 3.024e-06 | speech_ratio | -0.4422672 | 1.015e-05 |
| amplitude_kurtosis | 0.4122244 | 4.447e-05 | number_of_pauses | -0.3738093 | 0.0002421 |
| number_of_pauses | -0.4005278 | 7.617e-05 | amplitude_mav | -0.3416138 | 0.0008601 |
| total_phonation_time | -0.3871873 | 0.0001374 | total_phonation_time | -0.3212951 | 0.001791 |
| amplitude_mav | -0.2895547 | 0.005117 | total_power | -0.3045195 | 0.003164 |
| peak_frequency | -0.2145304 | 0.04002 | mean_power | -0.3045195 | 0.003164 |
| total_power | -0.199052 | 0.05714 | harmonics_to_noise_ratio | -0.3018382 | 0.003455 |
| mean_power | -0.199052 | 0.05714 | peak_frequency | -0.2815369 | 0.006554 |
| harmonics_to_noise_ratio | -0.1656924 | 0.1145 | amplitude_kurtosis | 0.2753473 | 0.007896 |
| amplitude_fourth_moment | -0.123723 | 0.24 | amplitude_fourth_moment | -0.250406 | 0.01606 |

(f) (Male;Apathy)

| **Positive story** |  |  | **Negative story** |  |  |
| --- | --- | --- | --- | --- | --- |
| **Feature** | **Correlation** | **p value** | **Feature** | **Correlation** | **p value** |
| speech_ratio | -0.5011488 | 0.0002446 | speech_ratio | -0.5768886 | 1.433e-05 |
| number_of_pauses | -0.3865379 | 0.00608 | amplitude_kurtosis | 0.455276 | 0.001013 |
| amplitude_kurtosis | 0.3169775 | 0.02648 | number_of_pauses | -0.4269726 | 0.002216 |
| total_phonation_time | -0.3050516 | 0.03306 | total_phonation_time | -0.3781761 | 0.00738 |
| amplitude_mav | -0.2994025 | 0.03662 | amplitude_mav | -0.2946426 | 0.03987 |
| peak_frequency | -0.2489267 | 0.08457 | peak_frequency | -0.2666586 | 0.06401 |
| total_power | -0.2485083 | 0.08511 | total_power | -0.2493975 | 0.08396 |
| mean_power | -0.2485083 | 0.08511 | mean_power | -0.2493975 | 0.08396 |
| espinola_zero_crossing_metric | -0.1929972 | 0.184 | local_jitter | 0.1978233 | 0.173 |
| mean_f0 | 0.1854857 | 0.202 | mean_f0 | 0.181538 | 0.2119 |

**TABLE S2.** Top 10 highest spearman rank partial correlations between audio features calculated per speech task and NPI subscales, corrected for MMSE. Results are split up by gender.

(a) (Female; Anxiety)

| **Positive story** |  |  | **Negative story** |  |  |
| --- | --- | --- | --- | --- | --- |
| **Feature** | **Correlation** | **p value** | **Feature** | **Correlation** | **p value** |
| sound_to_noise_ratio | 0.33073 | 0.00136 | mean_f0 | -0.36523 | 0.00037 |
| peak_frequency | -0.20172 | 0.05518 | peak_frequency | -0.35242 | 0.00061 |
| amplitude_fourth_moment | -0.17364 | 0.09975 | total_power | -0.32664 | 0.00158 |
| total_power | -0.17349 | 0.10004 | mean_power | -0.32664 | 0.00158 |
| mean_power | -0.17349 | 0.10004 | amplitude_mav | -0.32015 | 0.00198 |
| amplitude_kurtosis | 0.16728 | 0.11298 | amplitude_fourth_moment | -0.29205 | 0.00497 |
| amplitude_mav | -0.16361 | 0.12124 | local_absolute_jitter | 0.28813 | 0.00561 |
| mean_f0 | -0.12298 | 0.24548 | sound_to_noise_ratio | 0.27374 | 0.00865 |
| local_absolute_jitter | 0.11589 | 0.27400 | max_amplitude | -0.23753 | 0.02339 |
| max_amplitude | -0.11202 | 0.29043 | ppq5_jitter | 0.23121 | 0.02744 |

(b) (Male; Anxiety)

| **Positive story** |  |  | **Negative story** |  |  |
| --- | --- | --- | --- | --- | --- |
| **Feature** | **Correlation** | **p value** | **Feature** | **Correlation** | **p value** |
| sound_to_noise_ratio | 0.39219 | 0.00583 | sound_to_noise_ratio | 0.40730 | 0.00406 |
| espinola_zero_crossing_metric | -0.17880 | 0.22400 | amplitude_kurtosis | 0.27846 | 0.05531 |
| power_spectrum_ratio | 0.13428 | 0.36285 | mean_f0 | -0.23137 | 0.11358 |
| speech_ratio | 0.13293 | 0.36776 | amplitude_mav | -0.22492 | 0.12430 |
| ppq5_jitter | -0.11695 | 0.42858 | ppq5_jitter | -0.17848 | 0.22484 |
| total_power | -0.10299 | 0.48604 | peak_frequency | -0.16178 | 0.27196 |
| mean_power | -0.10299 | 0.48604 | number_of_pauses | -0.15929 | 0.27950 |
| mean_f0 | -0.10192 | 0.49062 | total_power | -0.15637 | 0.28852 |
| average_amplitude_change | -0.08493 | 0.56600 | mean_power | -0.15637 | 0.28852 |
| amplitude_fourth_moment | -0.07194 | 0.62704 | speech_ratio | -0.14818 | 0.31482 |

(c) (Female; Depression)

| **Positive story** |  |  | **Negative story** |  |  |
| --- | --- | --- | --- | --- | --- |
| **Feature** | **Correlation** | **p value** | **Feature** | **Correlation** | **p value** |
| peak_frequency | -0.18970 | 0.07170 | max_amplitude | -0.26266 | 0.01189 |
| amplitude_fourth_moment | -0.18571 | 0.07800 | amplitude_fourth_moment | -0.22337 | 0.03331 |
| total_power | -0.17313 | 0.10076 | peak_frequency | -0.22293 | 0.03367 |
| mean_power | -0.17313 | 0.10076 | total_power | -0.21372 | 0.04193 |
| max_amplitude | -0.14265 | 0.17737 | mean_power | -0.21372 | 0.04193 |
| amplitude_mav | -0.14058 | 0.18381 | amplitude_mav | -0.18209 | 0.08408 |
| amplitude_third_moment | -0.13400 | 0.20540 | amplitude_third_moment | -0.17726 | 0.09277 |
| mean_f0 | -0.12836 | 0.22530 | local_jitter | 0.15316 | 0.14722 |
| sound_to_noise_ratio | 0.12835 | 0.22533 | local_absolute_jitter | 0.14442 | 0.17200 |
| average_amplitude_change | -0.09983 | 0.34644 | ppq5_jitter | 0.13458 | 0.20342 |

(d) (Male; Depression)

| **Positive story** |  |  | **Negative story** |  |  |
| --- | --- | --- | --- | --- | --- |
| **Feature** | **Correlation** | **p value** | **Feature** | **Correlation** | **p value** |
| harmonics_to_noise_ratio | 0.22161 | 0.13008 | average_amplitude_change | 0.28733 | 0.04768 |
| average_amplitude_change | 0.19633 | 0.18108 | amplitude_fourth_moment | 0.14453 | 0.32705 |
| local_jitter | -0.17628 | 0.23070 | local_absolute_jitter | -0.13237 | 0.36979 |
| ppq5_jitter | -0.15358 | 0.29733 | peak_frequency | 0.12938 | 0.38080 |
| rap_jitter | -0.13196 | 0.37127 | speech_ratio | 0.11710 | 0.42801 |
| ddp_jitter | -0.13196 | 0.37127 | power_spectrum_ratio | 0.10835 | 0.46352 |
| local_absolute_jitter | -0.12744 | 0.38802 | number_of_pauses | 0.10633 | 0.47196 |
| amplitude_fourth_moment | 0.09941 | 0.50142 | amplitude_kurtosis | -0.09845 | 0.50561 |
| power_spectrum_ratio | 0.09347 | 0.52743 | sound_to_noise_ratio | 0.09809 | 0.50713 |
| max_amplitude | 0.09334 | 0.52804 | total_power | 0.08377 | 0.57134 |

(e) (Female; Apathy)

| **Positive story** |  |  | **Negative story** |  |  |
| --- | --- | --- | --- | --- | --- |
| **Feature** | **Correlation** | **p value** | **Feature** | **Correlation** | **p value** |
| amplitude_kurtosis | 0.22271 | 0.03385 | harmonics_to_noise_ratio | -0.24608 | 0.01871 |
| speech_ratio | -0.19707 | 0.06116 | speech_ratio | -0.22413 | 0.03270 |
| total_phonation_time | -0.17709 | 0.09311 | ppq5_jitter | 0.18561 | 0.07816 |
| number_of_pauses | -0.16877 | 0.10978 | rap_jitter | 0.17773 | 0.09191 |
| sound_to_noise_ratio | -0.16037 | 0.12889 | ddp_jitter | 0.17773 | 0.09191 |
| harmonics_to_noise_ratio | -0.12995 | 0.21955 | local_jitter | 0.16416 | 0.11998 |
| mean_f0 | 0.10509 | 0.32151 | sound_to_noise_ratio | -0.13812 | 0.19168 |
| amplitude_mav | -0.05878 | 0.57995 | number_of_pauses | -0.13435 | 0.20419 |
| ppq5_jitter | 0.05321 | 0.61643 | espinola_zero_crossing_metric | -0.11075 | 0.29597 |
| power_spectrum_ratio | -0.05298 | 0.61795 | total_phonation_time | -0.10475 | 0.32307 |

(f) (Apathy, Male)

| **Positive story** |  |  | **Negative story** |  |  |
| --- | --- | --- | --- | --- | --- |
| **Feature** | **Correlation** | **p value** | **Feature** | **Correlation** | **p value** |
| speech_ratio | -0.51070 | 0.00021 | speech_ratio | -0.51766 | 0.00016 |
| amplitude_mav | -0.33569 | 0.01967 | number_of_pauses | -0.35981 | 0.01201 |
| number_of_pauses | -0.30012 | 0.03821 | total_phonation_time | -0.35587 | 0.01305 |
| total_power | -0.29197 | 0.04405 | amplitude_kurtosis | 0.35308 | 0.01383 |
| mean_power | -0.29197 | 0.04405 | peak_frequency | -0.27395 | 0.05954 |
| peak_frequency | -0.27980 | 0.05409 | amplitude_mav | -0.26857 | 0.06493 |
| amplitude_kurtosis | 0.26409 | 0.06971 | total_power | -0.25654 | 0.07840 |
| total_phonation_time | -0.22924 | 0.11704 | mean_power | -0.25654 | 0.07840 |
| amplitude_fourth_moment | -0.20050 | 0.17180 | amplitude_fourth_moment | -0.19747 | 0.17852 |
| average_amplitude_change | -0.19865 | 0.17588 | amplitude_third_moment | -0.17273 | 0.24038 |

**TABLE S3.** Top 5 highest feature weights selected by SVM regression models in predicting each NPI subscale, seperated for females and males.

1. Anxiety

| **Female** | | | |  | **Male** | | | |
| --- | --- | --- | --- | --- | --- | --- | --- | --- |
| **Feature** | **Task** | **Mean** | **Standard Deviation** |  | **Feature** | **Task** | **Mean** | **Standard Deviation** |
| MFCC 18 | Neg | 0.0192 | 0.00076 |  | MFCC 9 | Neg | 0.0170 | 0.00050 |
| MFCC 4 | Neg | 0.0188 | 0.00061 |  | MFCC 4 | Pos | 0.0154 | 0.00060 |
| Sound-to-noise ratio | Pos | 0.0182 | 0.00070 |  | MFCC 10 | Pos | 0.0146 | 0.00051 |
| Deltas 7 | Pos | 0.0178 | 0.00083 |  | MFCC 16 | Neg | 0.0143 | 0.00075 |
| Delta Deltas 17 | Neg | 0.0172 | 0.00086 |  | MFCC 6 | Neg | 0.0141 | 0.00067 |

1. Depression

| **Female** | | | |  | **Male** | | | |
| --- | --- | --- | --- | --- | --- | --- | --- | --- |
| **Feature** | **Task** | **Mean** | **Standard Deviation** |  | **Feature** | **Task** | **Mean** | **Standard Deviation** |
| Delta Deltas 11 | Neg | 0.0014 | 5.13151e-05 |  | Delta Deltas 5 | Neg | 0.0009 | 6.46563e-05 |
| Deltas 8 | Pos | 0.0013 | 6.30781e-05 |  | Delta Deltas 20 | Neg | 0.0009 | 9.55432e-05 |
| Deltas 9 | Pos | 0.0011 | 5.65088e-05 |  | Amplitude kurtosis | Neg | 0.0008 | 3.10521e-05 |
| Deltas 16 | Neg | 0.0009 | 5.16828e-05 |  | Deltas 1 | Neg | 0.0007 | 7.12870e-05 |
| Deltas 17 | Neg | 0.0009 | 6.49522e-05 |  | Delta deltas 14 | Pos | 0.0007 | 7.22985e-05 |

(c) Apathy

| **Female** | | | |  | **Male** | | | |
| --- | --- | --- | --- | --- | --- | --- | --- | --- |
| **Feature** | **Task** | **Mean** | **Standard Deviation** |  | **Feature** | **Task** | **Mean** | **Standard Deviation** |
| MMSE |  | 0.0035 | 0.00011 |  | Speech ratio | Neg | 0.2203 | 0.09936 |
| Speech ratio | Pos | 0.0021 | 0.00011 |  | MMSE |  | 0.1910 | 0.09611 |
| Speech ratio | Neg | 0.0019 | 0.00012 |  | Speech ratio | Pos | 0.1774 | 0.04123 |
| Harmonics-to-noise ratio | Neg | 0.0018 | 9.59043e-05 |  | Deltas 5 | Neg | 0.1289 | 0.09128 |
| Deltas 2 | Pos | 0.0017 | 8.55444e-05 |  | Number of pauses | Neg | 0.1187 | 0.02705 |
